# Supplementary material for: The Divergent Key Residues of Two Agrobacterium fabrum (tumefaciens) CheY Paralogs Play a Key Role in Distinguishing Their Functions
Source: Microorganisms. 2021 May 24;9(6):1134. doi: 10.3390/microorganisms9061134 (PMC8225110; doi:10.3390/microorganisms9061134)
Supplement: Supplementary file 1 [file microorganisms-09-01134-s001.zip › microorganisms-1221598-supplementary.pdf]

microorganisms

Supplementary Data

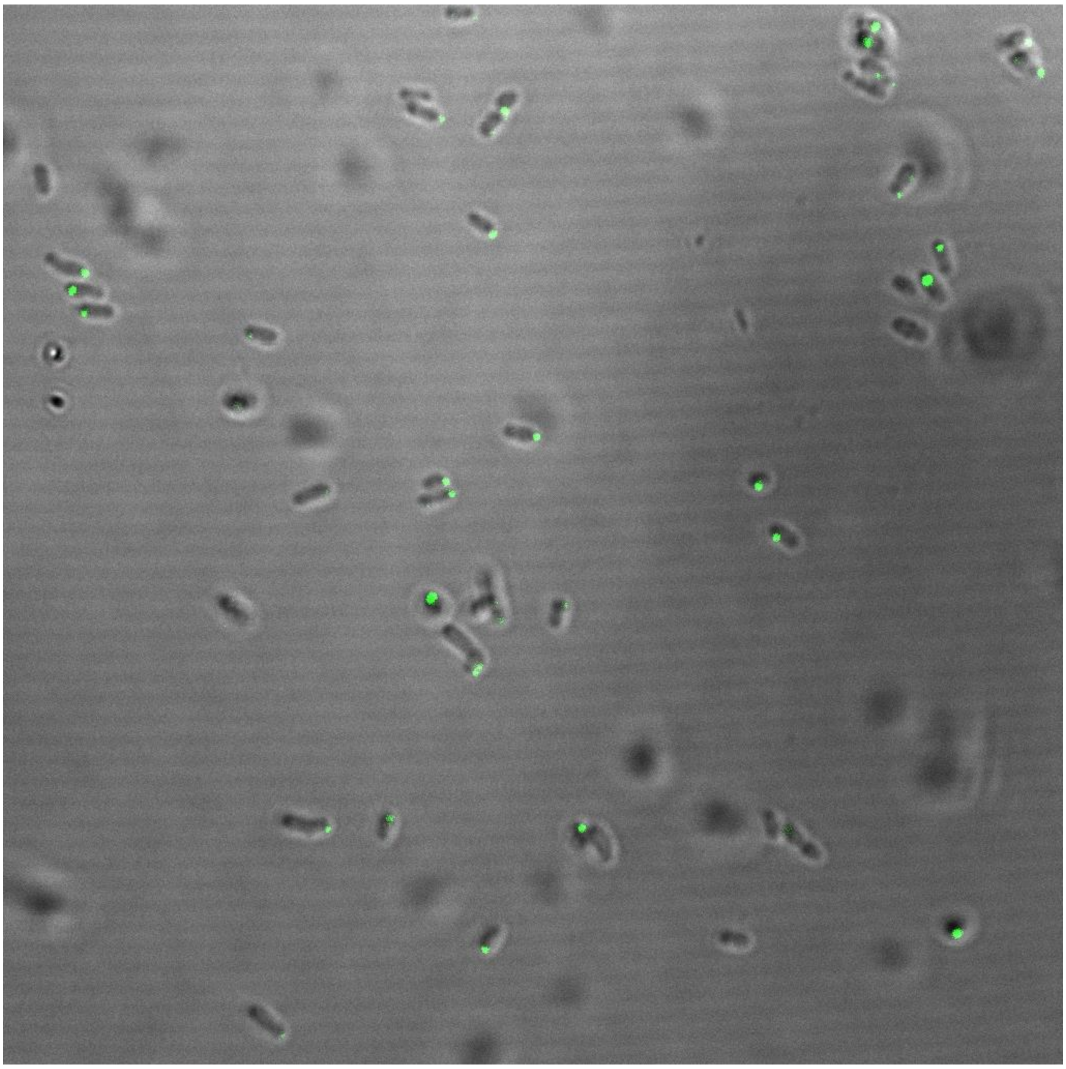

Figure S1. Polar co-localization of CheA with CheW1. eGFP was split into two non-fluorescent parts. The C-terminal part (Cegfp) of eGFP was fused to the C-terminus of CheA and the N-terminal part (Negfp) of eGFP was fused to the C-terminus of CheW1. CheA-Cegfp and CheW1-Negfp fusion proteins were equivalently co-expressed *in cheA-cheW1-cheW2* triple-deletion mutant (Δaw). Interaction between two fusion proteins will result in the reconstitution of two split-eGFP parts and the reconstituted eGFP will emit fluorescence. Cells of mutant Δaw expressing these two fusion proteins were observed by using confocal laser-scanning microscopy.

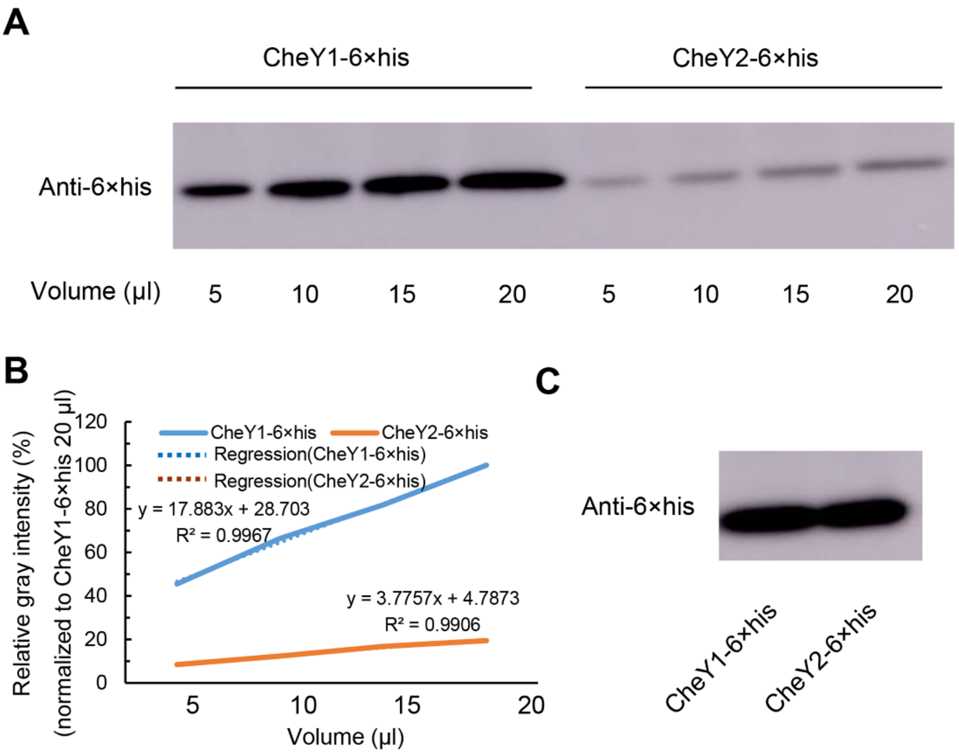

Figure S2. Estimation of the amount of CheY1-6×his and CheY2-6×his by Western blot. Different amounts of crude extraction of CheY1-6×his or CheY2-6×his were loaded to run SDS-PAGE. The separated proteins were analyzed by Western blot using anti-6×his tag antibody. (A) Western blotting band of CheY1-6×his and CheY2-6×his. The darkness of the Western blotting band is proportional to the amount of loaded CheY1-6×his or CheY2-6×his. (B) Relative gray intensity of the Western blotting band in (A). Relative gray intensity of the band is linear with the amount of loaded CheY1-6×his or CheY2-6×his and thus can be used to estimate the amount of CheY1-6×his or CheY2-6×his in the sample. (C) Western blotting determination of the relative concentration of CheY1-6×his and CheY2-6×his in their respective crude extract before using for pull-down.

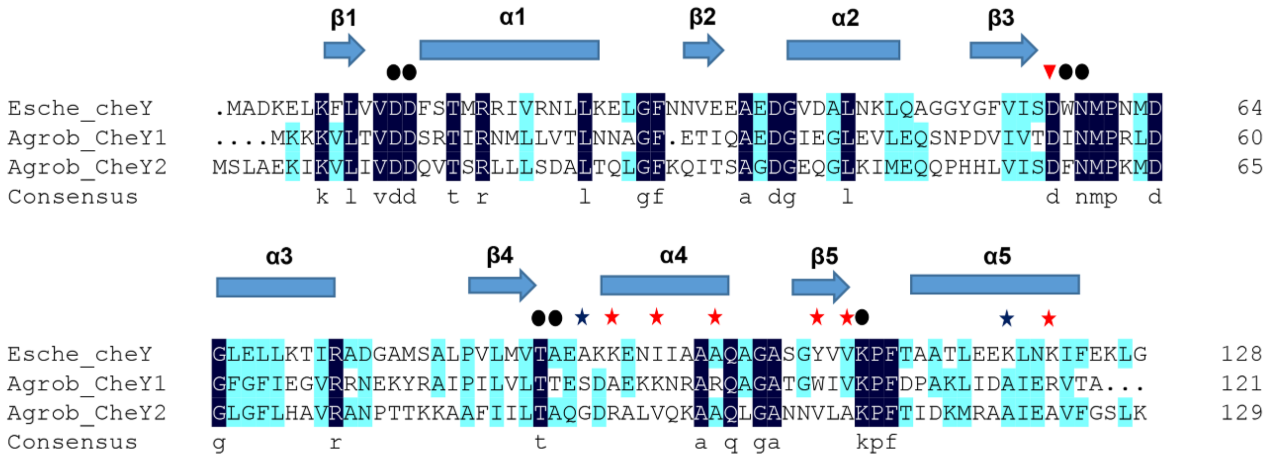

Figure S3. Sequence alignment of two CheYs of *Agrobacterium fabrum* with the CheY of *Escherichia coli*. Secondary structure elements of *E. coli* CheY are shown above the sequence (α represents α-helical structure and β represents β-folded structure). Numbers on the right indicate the position of amino acid. Sequences of two *A. fabrum* CheY proteins are from genome database (genome accession number: AE007869.2). Triangle indicates the active site residue of phosphorylation [54]. Black dots indicate the residues of the active-site pocket of phosphorylation in *E. coli* CheY [41]. Asterisks indicate the residues of *E. coli* CheY involved in FliM binding [42]. The red asterisks indicate the site-directed mutation positions constructed in this work.

Table S1. Bacterial strains and plasmids used in this study.

Bacterial strains and plasmids	Relevant feature(s)	Source or reference
**Strains**		
Escherichia coli		
DH5a	*EndA1 hsdR17 supE44 thi-1 recA1 gyrA96 relA1* (*argF-lacZYA*) U169 φ80d*lacZ*, for DNA cloning	Bethesda Research Laboratories
BL21(DE3)	*F⁻ ompT hsdSB(rB⁻mB⁻) gal dcm (DE3)*, for protein expression	Invitrogen
XL1 Blue	Report strain for Bacterial Two-hybrid System	Stratagene
Agrobacterium fabrum		
C58	Nopaline type strain; pTiC58, pAtC58	Thomashow *et al.* [55]. Knauf and Nester [56]
Δy1	Derivative of C58 in which *cheY1* (*atu0516*) open reading frame (ORF) was deleted	This study
Δy2	Derivative of C58 in which *cheY2* (*atu0520*) ORF was deleted	This study
Δy	Derivative of C58 in which both *cheY1* and *cheY2* ORFs were deleted	This study
Δy1+y1	Δy1 in which *cheY1* expression was restored by plasmid pUCA-Y1	This study

Δy2+y2	Δy2 in which *cheY2* expression was restored by plasmid pUCA-Y2	This study
Δy+y1	Δy in which *cheY1* expression was restored by plasmid pUCA-Y1	This study
Δy+y2	Δy in which *cheY2* expression was restored by plasmid pUCA-Y2	This study
Δa	Derivative of C58 in which *cheA* (*atu0517*) ORF was deleted	Huang *et al.* [27]
Δaw	Derivative of C58 in which *cheA*, *cheW1* and *cheW2* ORFs were deleted	Huang *et al.* [27]
Δay	Derivative of C58 in which *cheA*, *cheY1* and *cheY2* ORFs were deleted	This study
Δays	Derivative of C58 in which *cheA*, *cheY1*, *cheY2* and *cheS* ORFs were deleted	This study
**Plasmids**		
pEX18Km	Derivative of pEX18Tc in which Tc^r was replaced by *npt*III from pCB301; Km^r, Sur^s	Huang *et al.* [27]
pCB301-GFP	A minim binary vector plasmid carrying the GFP ORF; Km^r	Guo *et al.* [29]
pUCA-19	pUC19 carrying an agrobacterial replicon; Ap^r, Cr^r	Guo *et al.* [29]
pET30a	Expression vector; Km^r	Novagen
pGEX-4T-1	Expression vector; Ap^r, Cr^r	GE Healthcare
pUCA-Y1	pUCA-19, carrying 366 bp *cheY1* ORF at *Hin*dIII and *Eco*RI; Ap^r, Cr^r	This study
pUCA-Y1^{A88R}	pUCA-19, carrying 366 bp CheY1^{A88R} expression cassette at *Hin*dIII and *Eco*RI; Ap^r, Cr^r	This study
pUCA-Y1^{K91V}	pUCA-19, carrying 366 bp CheY1^{K91V} expression cassette at *Hin*dIII and *Eco*RI; Ap^r, Cr^r	This study
pUCA-Y1^{R95A}	pUCA-19, carrying 366 bp CheY1^{R95A} expression cassette at *Hin*dIII and *Eco*RI; Ap^r, Cr^r	This study
pUCA-Y1^{W102V}	pUCA-19, carrying 366 bp CheY1^{W102V} expression cassette at *Hin*dIII and *Eco*RI; Ap^r, Cr^r	This study
pUCA-Y1^{V104A}	pUCA-19, carrying 366 bp CheY1^{V104A} expression cassette at *Hin*dIII and *Eco*RI; Ap^r, Cr^r	This study
pUCA-Y1^{R118A}	pUCA-19, carrying 366 bp CheY1^{R118A} expression cassette at *Hin*dIII and *Eco*RI; Ap^r, Cr^r	This study
pUCA-Y2	pUCA-19, carrying 390 bp *cheY2* ORF at *Hin*dIII and *Eco*RI; Ap^r, Cr^r	This study
pUCA-Y2^{R93A}	pUCA-19, carrying 390 bp CheY2^{R93A} expression cassette at *Hin*dIII and *Eco*RI; Ap^r, Cr^r	This study
pUCA-Y2^{V96K}	pUCA-19, carrying 390 bp CheY2^{V96K} expression cassette at *Hin*dIII and *Eco*RI; Ap^r, Cr^r	This study
pUCA-Y2^{A100R}	pUCA-19, carrying 390 bp CheY2^{A100R} expression cassette at *Hin*dIII and *Eco*RI; Ap^r, Cr^r	This study

pUCA-Y2^{V107W}	pUCA-19, carrying 390 bp CheY2^{V107W} expression cassette at *Hin*dIII and *Eco*RI; Ap^r, Cr^r	This study
pUCA-Y2^{A109V}	pUCA-19, carrying 390 bp CheY2^{A109V} expression cassette at *Hin*dIII and *Eco*RI; Ap^r, Cr^r	This study
pUCA-Y2^{A123R}	pUCA-19, carrying 390 bp CheY2^{A123R} expression cassette at *Hin*dIII and *Eco*RI; Ap^r, Cr^r	This study
pEXY1	pEX18Km carrying a 979 bp fragment at *Bam*HI and *Xho*I, which consisted of 474 bp upstream of *cheY1* and 505 bp downstream of *cheY1*; Km^r	This study
pEXY2	pEX18Km carrying a 1,196 bp fragment at *Bam*HI and *Xho*I, which consisted of 596 bp upstream of *cheY2* and 600 bp downstream of *cheY2*; Km^r	This study
pEXS	pEX18Km carrying a 1,053 bp fragment at *Bam*HI and *Xho*I, which consisted of 563 bp upstream of *cheS* and 490 bp downstream of *cheS*; Km^r	This study
pGEX-FliM	pGEX-4T-1, carrying 960 bp *fliM* ORF at *Bam*HI and *Sma*I; Ap^r, Cr^r	This study
pET30a-CheY1	pET30a, carrying 363 bp *cheY1* ORF without stop codon at *Nde*I and *Xho*I; Km^r	This study
pET30a-CheY2	pET30a, carrying 387 bp *cheY2* ORF without stop codon at *Nde*I and *Xho*I; Km^r	This study
pUCA-SGAW1	pUCA19, carrying *cheA-Cegfp* and *cheW1-Negfp* expression cassette at *Hin*dIII and *Eco*RI; Ap^r, Cr^r	This study
pUCA-SGAY1	pUCA19, carrying *cheA-Cegfp* and *cheY1-Negfp* expression cassette at *Hin*dIII and *Eco*RI; Ap^r, Cr^r	This study
pUCA-SGAY2	pUCA19, carrying *cheA-Cegfp* and *cheY2-Negfp* expression cassette at *Hin*dIII and *Eco*RI; Ap^r, Cr^r	This study
pUCA-SGY12	pUCA19, carrying *cheY2-Cegfp* and *cheY1-Negfp* expression cassette at *Hin*dIII and *Eco*RI; Ap^r, Cr^r	This study
pUCA-SGY1	pUCA19, carrying *Cegfp* and *cheY1-Negfp* expression cassette at *Hin*dIII and *Eco*RI; Ap^r, Cr^r	This study
pUCA-SGAY1-Y2	pUCA19, carrying *cheA-Cegfp* , *cheY1-Negfp*, and *cheY2* ORF expression cassette at *Hin*dIII and *Eco*RI; Ap^r, Cr^r	This study
pUCA-SGAY2-Y1	pUCA19, carrying *cheA-Cegfp* , *cheY2-Negfp* and *cheY1* ORF expression cassette at *Hin*dIII and *Eco*RI; Ap^r, Cr^r	This study

Ap^r, Cr^r, Km^r, and Tc^r = Resistant to ampicillin, carbenicillin, kanamycin, and tetracycline, respectively; Sur^s = sucrose sensitivity; ORF = open reading frame.

Table S2. Primers used in this study.

Primers	Sequence	Purpose
dY1-1	5'-GACTCTAGAGGATCCCAGCGCCTCCGAGGCCG-3'	To amplify the upstream sequence of *cheY1*
dY1-2	5'-TCGTGAAATGTCCCGTATCACTTTTGCATCTCCT-3'	To amplify the upstream sequence of *cheY1*
dY1-3	5'-TACGGGACATTTCACGATGGATATGAACGAAATC-3'	To amplify the downstream sequence of *cheY1*
dY1-4	5'-TGCTGCCAACTCGAGCGGCTCGAAACCGCTTT-3'	To amplify the downstream sequence of *cheY1*
dY2-1	5'-GACTCTAGAGGATCCGGCGGGTCGTAAGGTCGTC-3'	To amplify the upstream sequence of *cheY2*
dY2-2	5'-GCCGCAGCTTCCATCATTTAGTCAGCACCTTCTTTGCG-3'	To amplify the upstream sequence of *cheY2*
dY2-3	5'-GAAGGTGCTGACTAAATGATGGAAGCTGCGGCC-3'	To amplify the downstream sequence of *cheY2*
dY2-4	5'-TGCTGCCAACTCGAGCGGAGTGGTGGCGGTGTG-3'	To amplify the downstream sequence of *cheY2*
dS-1	5'-GACTCTAGAGGATCCTTCCAGACCAACCTTCTCG-3'	To amplify the upstream sequence of *cheS*
dS-2	5'-TTACATGACTCCCTGACGTC-3'	To amplify the upstream sequence of *cheS*
dS-3	5'-CAGGGAGTCATGTAAATGGATATGAACGAAATC-3'	To amplify the downstream sequence of *cheS*
dS-4	5'-TGCTGCCAACTCGAGCGGCTCGAAACCGCTTT-3'	To amplify the downstream sequence of *cheS*
pUCAY1-f	5'-GATTACGCCAAGCTTGGTGAAGAAAAAGTTCT-3'	To amplify the sequence of *cheY1* inserting pUCA19
pUCAY1-r	5'-ACGGCCAGTGAATTCTCAGGCGGTTACGCGCT-3'	To amplify the sequence of *cheY1* inserting pUCA19
pUCAY2-f	5'-GATTACGCCAAGCTTGATGTCTCTCGCAGAAAA-3'	To amplify the sequence of *cheY2* inserting pUCA19
pUCAY2-r	5'-ACGGCCAGTGAATTCTCATTTCAGCGATCCGA-3'	To amplify the sequence of *cheY2* inserting pUCA19
SGA-f	5'-GATTACGCCAAGCTTGATGGATATGAACGAAATC-3'	To amplify the sequence of *cheA* fusing to *Cegfp*

SGA-r	5'-TCCACCCGACGTCCCAAGCTTACCCGTCGCCGCGAGTG-3'	To amplify the sequence of *cheA* fusing to *Cegfp*
SGW1-f	5'-TTCGAGGATGCGACTATGTCCAACGCCATCAA-3'	To amplify the sequence of *cheW1* fusing to *Negfp*
SGW1-r	5'-AGAGCCAGAGCCACCGGCCGCTTCGCGCGCCA-3'	To amplify the sequence of *cheW1* fusing to *Negfp*
gfp-1	5'-GGTGGCTCTGGCTCTGGCTCGAGGGTGAGCAAGGGCGAGGA-3'	To amplify the N-terminal sequence of *egfp*
gfp-2	5'-ACGGCCAGTGAATTCTTACTGCTTGTCGGCCATGA-3'	To amplify the N-terminal sequence of *egfp*
gfp-3	5'-GGGACGTCGGGTGGAAGCGGTAAGAACGGCATCAAGGTG-3'	To amplify the C-terminal sequence of *egfp*
gfp-4	5'-AGTCGCATCCTCGAATTCTTACTTGTACAGCTCGTC-3'	To amplify the C-terminal sequence of *egfp*
SGY2-1	5'-TTCGAGGATGCGACTATGTCTCTCGCAGAAAA-3'	To amplify the sequence of *cheY2* fusing to *Negfp*
SGY2-2	5'-AGAGCCAGAGCCACCTTTCAGCGATCCGAAAAC-3'	To amplify the sequence of *cheY2* fusing to *Negfp*
SGY2-3	5'-GATTACGCCAAGCTTGATGTCTCTCGCAGAAAA-3'	To amplify the sequence of *cheY2* fusing to *Cegfp*
SGY2-4	5'-TCCACCCGACGTCCCAAGCTTTTTCAGCGATCCGAAAAC-3'	To amplify the sequence of *cheY2* fusing to *Cegfp*
SGY1-f	5'-TTCGAGGATGCGACTGTGAAGAAAAAAGTTCT-3'	To amplify the sequence of *cheY1* fusing to *Negfp*
SGY1-r	5'-AGAGCCAGAGCCACCGGCGGTTACGCGCTCAA-3'	To amplify the sequence of *cheY1* fusing to *Negfp*
Y1^{A88R}-f	5'-GATCGGGAAAAGAAGAACCGCGC-3'	To amplify the sequence of pUCA-Y1^{A88R}
Y1^{A88R}-r	5'-GCTTTCGGTCGTCAGAAC-3'	To amplify the sequence of pUCA-Y1^{A88R}
Y1^{K91V}-f	5'-AAGGTGAACCGCGCCCGCCAGG-3'	To amplify the sequence of pUCA-Y1^{K91V}
Y1^{K91V}-r	5'-TTCCGCATCGCTTTCGGT-3'	To amplify the sequence of pUCA-Y1^{K91V}
Y1^{R95A}-f	5'-GCCCAGGCCGGTGCGACCG-3'	To amplify the sequence of pUCA-Y1^{R95A}
Y1^{R95A}-r	5'-GGCGCGGTTCTTCTTTTCC-3'	To amplify the sequence of pUCA-Y1^{R95A}
Y1^{W102V}-f	5'-GGCGTGATCGTCAAGCCGTTCGA-3'	To amplify the sequence of pUCA-Y1^{W102V}

Y1^{W102V}-r	5'-GGTCGCACCGGCCTGGC-3'	To amplify the sequence of pUCA-Y1^{W102V}
Y1^{V104A}-f	5'-ATCGCCAAGCCGTTCGACCCTGC-3'	To amplify the sequence of pUCA-Y1^{V104A}
Y1^{V104A}-r	5'-CCAGCCGGTCGCACCGG-3'	To amplify the sequence of pUCA-Y1^{V104A}
Y1^{R118A}-f	5'-GAGGCCGTAACCGCCTGAGAAT-3'	To amplify the sequence of pUCA-Y1^{R118A}
Y1^{R118A}-r	5'-AATGGCATCGATGAGTTT-3'	To amplify the sequence of pUCA-Y1^{R118A}
Y2^{R93A}-f	5'-GCCGCGCTGGTGCAGAAGGC-3'	To amplify the sequence of pUCA-Y2^{R93A}
Y2^{R93A}-r	5'-GTCACCCTGCGCGGTGAG-3'	To amplify the sequence of pUCA-Y2^{R93A}
Y2^{V96K}-f	5'-CTGAAGCAGAAGGCAGCCCAGCT-3'	To amplify the sequence of pUCA-Y2^{V96K}
Y2^{V96K}-r	5'-CGCGCGGTCACCCTGCG-3'	To amplify the sequence of pUCA-Y2^{V96K}
Y2^{A100R}-f	5'-CGCCAGCTCGGCGCCAACAA-3'	To amplify the sequence of pUCA-Y2^{A100R}
Y2^{A100R}-r	5'-TGCCTTCTGCACCAGCGC-3'	To amplify the sequence of pUCA-Y2^{A100R}
Y2^{V107W}-f	5'-AACTGGCTGGCCAAGCCCTTCA-3'	To amplify the sequence of pUCA-Y2^{V107W}
Y2^{V107W}-r	5'-GTTGGCGCCGAGCTGGG-3'	To amplify the sequence of pUCA-Y2^{V107W}
Y2^{A109V}-f	5'-CTGGTCAAGCCCTTCACCATCGA-3'	To amplify the sequence of pUCA-Y2^{A109V}
Y2^{A109V}-r	5'-CACGTTGTTGGCGCCGAG-3'	To amplify the sequence of pUCA-Y2^{A109V}
Y2^{A123R}-f	5'- GAACGGGTTTTCGGATCGCTGAA-3'	To amplify the sequence of pUCA-Y2^{A123R}
Y2^{A123R}-r	5'-GATGGCCGCGCGCATCTT-3'	To amplify the sequence of pUCA-Y2^{A123R}

Reference

54 Sanders, D.A.; Gillece-Castro, B.L.; Stock, A.M.; Burlingame, A.L.; Koshland, D.E. Identification of the site of phosphorylation of the chemotaxis response regulator protein, CheY. *J. Biol. Chem*. **1989**, 264, 21770-21778.

55. Thomashow, M.F.; Nutter, R.; Montoya, A.L.; Gordon, M.P.; Nester, E.W. Integration and organization of Ti plasmid sequences in crown gall tumors. *Cell* **1980**, 19, 729-739.

56. Knauf, V.C.; Nester, E.W. Wide host range cloning vectors: A cosmid clone bank of an *Agrobacterium* Ti plasmid. *Plasmid* **1982**, 8, 45-54.
